# The Tropics and Life Assurance

**Published:** 1911-02-25

**Authors:** 


### The Hospital

A Journal of

The Medical Sciences and Hospital Administration.

New Series. No. 212, Vol. VIII. [No. 1284, Vol XLIX.] SATURDAY, FEBRUARY 25, 1911.

### The Tropics and Life Assurance.

With the progress of tropical medicine—and especially of its preventive phases—everyone in the medical profession is anxious and eager to keep abreast; and it is indeed becoming widely diffused among the general community that the hygiene of tropical life is a science which is steadily making up its centuries of leeway. There is an aspect of life in the tropics, however, with regard to which modern discoveries and modern theories seem to have effected little if any change, to wit the effect of a tropical residence on the proposal of a European for life insurance. The lethargic indifference of the insurance offices to the numerous important points in this connection which are deserving of their attentive study has on more than one occasion moved Mr. Cantlie to expressions of opinion, and he has recently again entered at length into the subject in a most complete and instructive paper.*

The question is, as will readily be comprehended, very complicated, and there are many factors to take into account. But Mr. Cantlie has exposed completely the fallacies and injustices of the present haphazard system of "loads," and it is hard to believe that the more progressive offices will make no attempts at improvement. The possibility has, no doubt, to be reckoned with that the offices do not care about risks whose magnitude they cannot precisely estimate, and stick to their absurd and antiquated tariffs as the readiest means of discouraging proposals from those who have to dwell in the tropics. Even then, the enterprising office which should first reform its practice would probably reap a golden harvest. One of the maddest of all the insurance companies' rules is that which insists on extra rates for those who live between 33° North latitude and 30° South. For there are health resorts close to the Equator—notably the highlands of British East Africa, some of the West Indies, and some of the Pacific Islands, where the climate is almost if not quite as good as that of Britain; whereas there are places outside those limits where the climate is more deadly and more truly tropical than at most places within them. To lump in Bermuda with the Upper Nile, or Johannesburg with Beira, as regions of equal salubrity for insurance purposes is a crazy perversity which should only require mention to be rectified. Yet there is a conspiracy of immobility among the offices, to whom the facts have repeatedly been represented.

Another large class of anomaly is provided by the influence of age upon tropical disease. It is a commonplace that the boy of eighteen or twenty who goes to a tropical place of bad reputation is far more likely to go down than the man of thirty, who in his turn has less chance of immunity than the healthy man of forty. Mr. Cantlie estimates the vulnerability of the first as ten times that of the last. For insurance purposes this is most unfortunate. The young man, whose salary is most likely to be small, can ill afford the increase of premiums, which is yet more required in his case than in that of his senior: the latter, on the other hand, can generally well afford the "load," but in strict justice should really not have to pay nearly so much, since the office is accepting a much smaller risk. But there are far stranger anomalies than this. It has happened that patients in temperate zones have been ordered by their physicians to warm climates, and have had to pay their offices extra rates for going there. In other words, they had to pay for the privilege of going to the only place where their lives would be prolonged! Again, in some cases men who have lived long in the tropics have been allowed to pay ordinary rates on returning sound to England. When the cold climate has proved too much for them, and they have returned to a warmer one, the load has been put on again. Here the extra premium has been rebated for the only climate the insurer's health could not stand!

What, then, has Mr. Cantlie to offer instead of the blundering unfairness of the present system? He suggests that after seven years' residence in a proscribed region the extra premium should be reduced one half, conditionally on the company's medical officer reporting at the insured's expense that no permanent damage to health has been sustained. This, by the way, assumes that the insured is a visitor to England at intervals; failing that the companies would have to appoint reputable referees at important tropical centres. Then after ten years the premium would be cut down to a third, and later to a quarter, conditionally as before. After fourteen years the premium would be normal. This still fails to get over the difficulty of unduly handicapping the young men who can least afford big premiums; and an alternative scheme suggested does the same thing in reality, though the author does not seem to see that it is so. In fact, no scheme can be just to the older men and not burdensome to the younger ones; because the former are better "lives." The present method makes the old men pay for the young ones. There is certainly a great deal in one of the ideas propounded: that of establishing a scale of unhealthiness for different places in the tropics. Thus Mauritius and British Guiana are rated as only two-thirds as salubrious as Ceylon or Hong Kong. But the practical difficulties would still be enormous. What office, for example, could distinguish thus between the ideal climate of Nairobi and the fever dens of Lamu, Mälindi, and other coast towns of the same Protectorate? Yet a soldier or civil servant may live for years at one and then be transferred to the other at a moment's notice, or may have to spend half the year in a health resort and the other half in a swamp. This is no argument against attempting to rectify those patent injustices which can be put right; and it is to be hoped Mr. Cantlie's protest may have some effect upon the hitherto recalcitrant offices.

**Journal of Tropical Medicine and Hygiene*, February 1, 1911.

